# Effects of routine physiotherapy with and without neuromobilization in the management of internal shoulder impingement syndrome: A randomized controlled trial

**DOI:** 10.12669/pjms.36.4.1545

**Published:** 2020

**Authors:** Muhammad Akhtar, Hossein Karimi, Syed Amir Gilani, Ashfaq Ahmad

**Affiliations:** 1 Dr. Muhammad Akhtar, M.Phil-PT. Department of Physiotherapy, Social Security Hospital, Gujranwala, Pakistan; 2 Prof. Hossein Karimi, Ph.D-PT. University Institute of Physiotherapy, The University of Lahore, Lahore, Pakistan; 3 Prof. Syed Amir Gilani, Ph.D Swiss. Dean Faculty of Allied Health Sciences, The University of Lahore, Lahore, Pakistan; 4 Dr. Ashfaq Ahmad, Ph.D-PT. University Institute of Physiotherapy, The University of Lahore, Lahore, Pakistan

**Keywords:** Pain, Rotator cuff, Shoulder Impingement Syndrome

## Abstract

**Background & Objective:**

Routine physiotherapy has been advocated was an effective treatment for internal shoulder impingement syndrome. However, there is lack of best exercise treatment and lots of studies are under consideration. The objective of the study was to compare the effects of Neuromobilization and routine physiotherapy on pain in patients having shoulder internal impingement syndrome.

**Methods:**

This is a single blinded randomized control clinical trial that was conducted at Social Security Hospital Gujranwala in which 80 patients with SIS were participated. The duration of study was from September 2016 to March 2018. Patients were recruited after giving an informed consent and were randomly assigned to either control or experimental group which was treated with routine physiotherapy and routine physiotherapy plus neuromobilization respectively; pain was assessed by Numeric Rating Scale at base line, 5^th^ and 11^th^ week.

**Results:**

The experimental group compared with control group at 11^th^ week had lower mean pain score 2.15(1.66-2.64) vs 4.90(4.41-5.40); between group difference, 1.82; 95% (CI), -2.38 to -1.25; P < 0.001 and Partial Ŋ^2^=0.33. These results show that pain score is much improved in experimental group.

**Conclusion:**

Neuromobilization along with physical therapy is more effective as compared to physiotherapy alone.

## INRODUCTION

The internal shoulder impingement syndrome (SIS) consists of the rotator cuff tendonitis and bursitis of the shoulder.^1^ The internal SIS involves the inflammation of the supraspinatus tendon between the anteroinferior junction of the acromion and the greater tuberosity of the humerus. SIS is categorized by severe pain that increases during overhead activities and at night sleeping on affected side.^2^

The internal SIS comprises of three stages, stage I impingement is defined by edema and hemorrhage of the subacromial bursa and rotator cuff, it is found in patients who are less than 25 years old. Stage II impingement represents irreversible changes, such as fibrosis and tendinopathy of the rotator cuff. It is mostly found in patients who are up to 25 to 40 years old. Stage III impingement is marked by more-chronic changes, such as partial or complete tears of the rotator cuff, and usually it is seen among the patients who are more than 40 years old.^3^

The concept of neuromobilization (NM) includes connection between mechanics and physiology of the nervous system in which interactions occur both ways and can be beneficial intensely for shoulder pain. Mechanical management may therefore be used to augment physiology in the nervous system. NM sequencing is the performance of set of particular component body movements so as to produce specific mechanical events in the nervous system, according to that sequence of component movements.^4^ In a review article of Matocha MA et al. 2015, it has already been observed that there are three theories projected for the local etiological origin of tendon pain: 1-mechanical, 2-vascular and 3-neural.^5^

Mechanical and vascular theories are regularly used for the treatment of tendon pain. The neural component is over looked due to poor outcomes among patients with tendinopathy. Matocha MA et al. highlighted neural involvement in patients with tendon pain and discussed the role of NM for tendon pain.^5^ The utilization of neurodynamics may be important for the treatment in patients who suffer with tendonopathies which has neural component.^5^,^6^

The neural theory includes number of elements that may lead to tendon pathology, increased stress and tension on nerve will result in decreased blood flow to the specific nerve which will affect blood flow to tendon.^7^ The proofs regarding advantages and disadvantages of rehabilitation of nerve related neck arm pain are required immediately. Neuromobilization is one of the physiotherapy treatment recommended for neck and arm pain to mitigate nerve sensitivity.^8^ The objective of the study was to compare the effects of NM technique and routine physiotherapy on pain in patients having shoulder internal impingement syndrome and to discover evidence based conservative and cost effective remedy for shoulder internal impingement syndrome.

## METHODS

Clinically analyzed eighty patients of internal SIS who visited physiotherapy department were enrolled for this specific analysis. The duration of study was from September 2016 to Match 2018. The study was described to all patients and informed consent was taken from them. This very consent process and Form was approved by Institutional Review Board, University of Lahore (IRB-UOL-FAHS/318/2018, dated: 24, April, 2018) before the initiation of random selection. Those participants were recruited who attended physiotherapy department, Social Security Hospital Gujranwala.

### Trial Registration

IRCT20190121042445N1. It was registered retrospectively.

### Subject selection and sampling procedure

The design of study was single blinded randomized controlled clinical trial. Sample size calculation was derived from Yamany AA et al.’s study.^9^ The sample size estimation formula was implemented.^10^


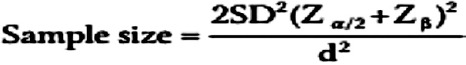


**Fig.1:**
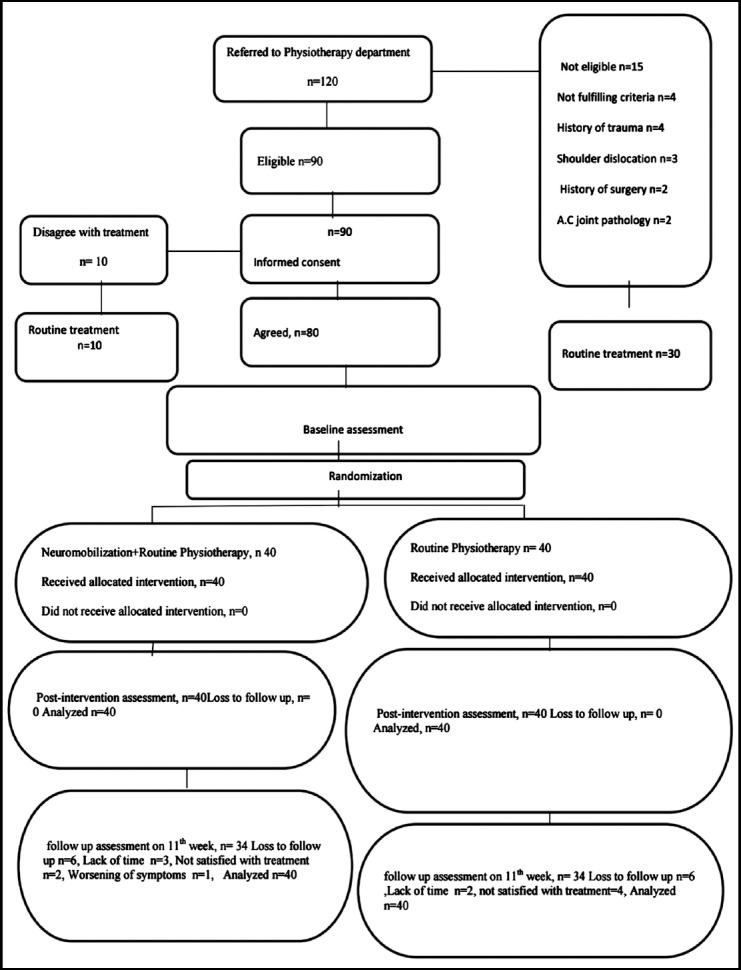
Flow sheet Diagram.

Where SD= Standard deviation=14.08, Z _1_-_α/2_ is type 1 error=1.96, Z_β_=0.84, d=µ_2-_µ_1_=10.7. Based on this a total sample size of around 80(experimental = 40, controls = 40) was calculated to be an adequate mean to reach the conclusion. Considering a loss of 20% follow-up, at least 80% patients followed the treatment.^11^ An experimental group and a control group were recruited which were based on the inclusion criteria for this study (Fig.1).

### Numeric Rating Scale

Numeric Rating Scale (NRS) was used to assess the intensity of pain which is valid and reliable measure of pain intensity. A continuous scale was used to ask the patients to think about their shoulder pain during the activity and to rate it by marking on a 10-mm line; it was anchored with “no pain” and the “worst pain you have ever felt”. This is a well-accepted method of evaluating the pain intensity levels.^12^

### Routine physiotherapy group

The routine Physiotherapy consisted of pulsed Short Wave Diathermy with frequency 27.12 MHZ, Ultrasonic Therapy with frequency 1.0 MHZ and intensity 1.45w/cm^2^,^13^ Transcutaneous Electrical Nerve Stimulator 2-200 HZ with output current < 20Ma width 200µ seconds along with continuous mode. Exercises comprised were shoulder strengthing and stretching exercises performed for 5 sec with 10 repetitions^14^ (Table-I).

**Table-I:** List of exercises performed under experimental and routine physiotherapy group.

Experimental group (stretching and strengthing exercises + Neuromobilization)	Routine physiotherapy group (Stretching and strengthing exercises)
***1)Stretching Exercises***	***1)Stretching Exercises***
a)Shoulder external rotation stretch	a)Shoulder external rotation stretch
b)Cross body posterior stretch	b)Cross body posterior stretch
c)Stretch for anterior aspect of shoulder	c)Stretch for anterior aspect of shoulder
d)Shoulder flexion stretch	d)Shoulder flexion stretch
***2)Strengthing Exercises***	***2)Strengthing Exercises***
a)Chair press	a)Chair press
b)Restricted scapular retraction	b)Restricted scapular retraction
c)Restricted scapular protraction	c)Restricted scapular protraction
d)Shoulder abduction “Scaption” (0°-90°) with theraband	d)Shoulder abduction “Scaption” (0°-90°) with theraband
e)Shoulder scapular extension with theraband	e)Shoulder scapular extension with theraband
***3)Neuromobilization Exercises***	
a)Neural slider technique	
b)Neural tensioner technique	

NM was applied using Butler’s recommendations.^15^ Initially, patient performed neural sliders and gradually progressed to neural tensioners. Neural sliders consisted of cervical lateral flexion movement, toward the involved side, simultaneously with elbow flexion and extension movements. While moving the head in to cervical lateral flexion the elbow was extended. When elbow began to flex, the cervical spine was returned to neutral position. Neural tensioners were performed to create tension in the nerve to get the desired results. The tension position was not held for a length of time, but was released by extending the elbow and returning the cervical spine to neutral. Once the patient had pushed slight pain or discomfort at any point,^16^ NM technique was performed for five seconds with 10 repetitions to control pain (Table-I). Both treatments were performed three times per week for total fifteen sessions over 05 weeks.

### Data Collection Methods

All participants had gone through a detailed systemic physical examination, that includes neurologic and musculoskeletal evaluations and the patients excluded from the above study who had gone through the shoulder surgery, shoulder injury, cervical radioculopathy and other systemic diseases. The patients who are diagnosed of having positive upper limb tension test^17^ along with Neer,^18^ Hawkins Kennedy^19^ Empty Can,^19^ painful arc test and cross body adduction test^20^ can undergo the specific treatment to get desired results. Randomization was performed by using computer generated random sequence table before the above assessment of the positive tests. Individuals, sequentially numbered index cards with random assignment were prepared for the study. The index cards were folded and placed in sealed and opaque envelopes. After baseline examination, the participants were randomly assigned to receive routine physiotherapy or routine physiotherapy combined with NM. One of the experienced staff members generated random allocation sequence.

An independent assessor, who specialized in musculoskeletal injuries with more than five year experience of dealing patients with shoulder injury, was masked to the group allocation of the patients for the treatment. The patients were evaluated at baseline, after last treatment (5^th^ week) and after 1^st^ follow up (11^th^ week).

All information was kept confidential. Participants remained anonymous throughout the study. They were informed that there were no disadvantages on procedure of study. They were being informed that they were free to withdraw at any time during the process of study.

### Data Analysis

The data were analyzed using SPSS version 22.0 software. Qualitative data were presented in frequencies and percentages. For quantitative data, mean and standard deviation (S.D) were calculated. Repeated measure ANOVA was applied among both the groups to calculate the average pain score at different times (baseline, 5^th^ week, 11^th^ week). The confidence interval of 95% was used and p value ≤ 0.05 was considered as significant.

## RESULTS

Comparison of demographic profile showed that most of the patients suffering from SIS were female 32 in experimental group and 26 in control group. It was also observed that mostly patients falling in type-1 Neer classification. The mean age of patients was 36.38±8.93 years in experimental group as compare to 34.40±9.32 years in control group shown in (Table-II).

**Table-II:** Comparison of Scio-demographic data of the patients.

Variable		Experimental Group (N=40)	Control Group (N=40)	P-Value
Age in Years		36.38±8.93	34.40±9.32	0.336
Gender	Male	8(20%)	14(32.4)	0.133
	Female	32(80%)	26(65%)	
Neer Test	Type 1: Pain at 90º	34(85.0%)	38(95.0%)	0.136
	Type 2: Pain at 60º-70º	6(15.0%)	2(5.0%)	
Pain/Discomfort on Palpation	A.C Joint	11(27.5%)	13(32.5%)	0.375
	S.C Joint	2(5.0%)	5(12.5%)	
	Biceps Tendon	27(67.5%)	22(55.0%)	

The results of pain are reported in (Table-III). For the control group, average shoulder pain was 6.78±1.04, and 4.90±1.58 at base line and 11^th^ week respectively. Similarly, average shoulder pain for experimental group (with NM) was 6.95±1.176, and 2.15±1.54 at base line and 11^th^ week respectively. Above mentioned results show the clear difference in average shoulder pain between two groups and shoulder pain of experimental group was more improved as compared to control group at different stages.

**Table-III:** Comparison of base line and final values for NRS across 2 treatment groups with P value.

Measurement	Group	Baseline value± SD	Final value on 11^th^ week± SD	P value
NRS	Control	6.78±1.04	4.9±1.58	<0.01
	Experimental	6.95±1.176	2.15±1.54	<0.01

There was a statistical significance with average difference=1.82, Partial Ŋ^2^=0.33 and p-value<0.001at 95% confidence interval in pain score at different time points (base line, 5^th^ week, 11^th^ week) between control and experimental group as shown in (Table-IV).

**Table-IV:** Comparison of Mean difference (95% CI) of between and within group comparison and Partial ŋ^2^ with P value.

Outcome Measures		Mean (95% CI)Within group Comparison		Mean Difference (95% Cl) of Between group Comparison by ANOVA (Experimental vs Control)	Partial ŋ^2^	P-value

		Experimental group	Control group			
Pain Assessment	Baseline	6.95(6.60-7.30)	6.78(6.42-7.13)	1.82(-2.38 to-1.25)	0.34	<0.001
	5^th^ week	2.15(1.60-2.71)	5.03(4.46-5.59)			
	11^th^ week	2.15(1.66-2.64)	4.90(4.41-5.40)			

## DISCUSSION

The results of present study demonstrated statistically significant differences in NRS scores between two groups. However, there was greater improvement in experimental group compared to control group. The findings of our study strengthen the fact that NM has beneficial effects.

The findings of the study of Pritam Deka prove the fact that NM has beneficial effects in reducing the pain by restoring neurodynamics properties in upper limb. Nee RJ et al. study has found immediate relief of pain in arm with no evidence of harmful effects and future research are recommended to check long term effects of NM on pain.^21^ Two studies on the use of mobilization have shown beneficial effects to control pain.^22^

The results of our study are also in agreement with Senbarsa G et al.^23^ who used manual treatment including deep frictional massage on supraspinatus muscle, radial nerve stretching, scapular mobilization, glenohumeral joint mobilization and proprioceptive neuromuscular facilitation techniques. These techniques in their study showed the effectiveness of manual therapy in supraspinatus tendinopathy. The use of manual therapy may help to relieve the pain.

The current study has found the results to be similar to those of Matocha et al. who found that pain intensity decreased weekly basis as decreased in our study on 5^th^ and 11^th^ week but further research is needed to help clinicians in making educational decisions for implementing these techniques in the clinical practice.^16^

From the available clinical research from the study of Mark T. Wash et al., it seems that a positive response is the provocation of patients’ pain, but there seems to be no clear indication for the appropriate duration, dosage, frequency, or type of exercise to be used; however, there is supporting evidence of NM as an effective treatment strategy.^17^

The use of neuromobilization produced clinically significant changes in patient’s status when other intervention options had failed to make any improvement in bench press and push-ups. Improvement of pain following NM may have occurred due to the involvement of different neurophysiological mechanisms.^24^ The reason for tissue repair is being observed in the study of Lederman E et al.^25^ In his study it was observed that normal tissue regeneration and remodeling depend on mechanical stimulation of nerve during the repair. This may help to enhance the tissue’s overall mechanical and physical behaviors, such as tensile strength and flexibility. Soft tissue and joint mobilization techniques have stimulated the more superficial level of proprioception, whereas the manual techniques of joint movement, stretching or deep kneading would stimulate the deep level of proprioception.

Different neuromuscular responses (like hypoalgesia, motor-neuron pool activity, afferent discharge and changes in the activity of muscle) indirectly associated with manual therapy indicates the spinal cord mediated effect of the manual therapy. Hypoalgesia following NM may also occur due to its effect mediated through spinal cord.^26^

Recognizing the close relationship between physical capacities and life style, it is likely that implementation of effective NM treatment as standard part of the treatment for SIS patients would decrease shoulder pain. This study shows that NM not only is feasible as part of the treatment, but also has a large effect size and is time efficient.

SIS patients suffer from many challenges, it is important to recognize that their shoulder pain constitutes an important part of overall health and daily tasks. Since SIS are known to be important key factor for daily life activities in term of pain. Importantly, this study, as well as NM regimes is feasible and safe to carry out within this patient group.

Su Y et al. highlights that neural tissue management is not better to other forms of treatment in decreasing pain.^27^ In another study it was also found that neuromobilization is no more useful or better treatment choice to lessen pain on nerve related chronic musculoskeletal conditions.^28^ However, it was also found that the lack of significance in pain between NM and other types of remedy may likely be due to lesser number of studies pooled; such that the meta-analysis was under powered to detect any true effect.^27^

### Limitation of the study

The patients included were collected from single hospital. They may have specific demographic and clinical features which might limit the generalization of the outcomes.

### Recommendations

According to published data summaries of research focusing on management of shoulder pain, it looks like that therapeutic exercise is not sufficient to treat shoulder internal impingement syndrome and it is compulsory to combine with other remedies to get the best results.^29^

### Rehabilitation implications

The outcomes of current study might be implicated that each of this outcome measure represented only one particular feature of clinical entity. To effectively treat a patient with internal SIS, the interventions require addressing the multiple aspects of the presenting clinical issues.

## CONCLUSION

NM technique is more effective and safe adjunct than routine physiotherapy in terms of greater reduction of pain in SIS patients.

### Authors’ Contribution

**MA:** Collected data, analysis and its interpretation.

**HK: C**onception & study design.

**PAG:** Revising it critically and final approval of manuscript.

**AA:** Drafting and data interpretation.

All authors have read and approved the manuscript.

The corresponding author Dr. Muhammad Akhtar is responsible and accountable for the accuracy or integrity of the work.
